# Study on the effect of Baduanjin online teaching on college students’ concentration

**DOI:** 10.3389/fspor.2026.1891811

**Published:** 2026-08-06

**Authors:** Lan Ma, Ruixuan Zhang, Zhiyuan Mao, Mu Han Teh

**Affiliations:** Department of Sports Sciences, college of Education, Zhejiang University, Hangzhou, China

**Keywords:** attention control scale (ACS), baduanjin, daily behavioral characteristics, focus, online intervention

## Abstract

Deteriorating attention control has become a common problem among contemporary college students caused by digital distractions, academic stress, and insufficient physical activity. As a mind-body exercise integrating mind, breath and movement, Baduanjin can improve cognitive function, yet the dose-dependent effects of online Baduanjin remain underexplored. This randomized controlled trial recruited 30 students (baseline ACS ≤50, <2 weekly exercise sessions with <5 h of weekly total activity) into high-frequency (7 sessions per week), low-frequency (3 sessions per week), and control groups for an 8-week intervention. Concentration was assessed via ACS scores, task initiation speed, and sustained attention duration. Both intervention groups outperformed controls on ACS; improvements in sustained-attention duration and initiation speed were markedly greater in the high-frequency group, whereas the low-frequency group showed significant gains only in initiation speed. High-frequency online Baduanjin effectively enhances college students' concentration and offers empirical support for integrating it into university extracurricular physical-education programs.

## 1 Introduction

Baduanjin is a classic traditional Chinese mind-body regulation exercise that integrates mental concentration, respiratory adjustment, and physical movement coordination. Its core feature is the unity of mind, breath, and posture, which enables systematic physical and psychological modulation. The declining concentration levels among college students have become a pressing is-sue, with approximately 50% of students maintaining sustained focus for merely 20 min during class sessions, significantly reducing learning efficiency (1, 2). This phenomenon primarily stems from digital distractions (such as checking phones every 6–8 min, which causes attention fragmentation), chronic stress caused by fast-paced lifestyles and performance pressures (which impair prefrontal cortex function), along with insufficient sleep and lack of physical activity. Exercise has a positive effect on improving concentration (3). It can enhance the function of the frontal lobe, promote the secretion of relevant neurotransmitters, improve cognitive control ability, and low-interference exercise can also help the brain recover from “directional attention fatigue” (4). As a traditional qigong, Baduanjin requires the coordination of movements, breathing and consciousness during practice, which can exercise the function of the brain' nervous system, regulate the endocrine system, and then improve concentration ability. Prior randomized controlled trials have verified that online mind-body intervention can improve university students' attentional abilities (5). Remote Baduanjin intervention has also been proven feasible within randomized controlled trial 38 frameworks (6). However, at present, most of the research on Baduanjin is an offline intervention, and most of it is used for clinical patient rehabilitation. There is almost no research on online intervention and the impact of attention on adults (especially college students).

This study set two explicit research hypotheses and a clear research objective in advance. The primary research objective was to systematically explore the dose-dependent effects of 8-week online Baduanjin practice frequencies (high-frequency vs. low-frequency) on improving college students' attention control. Hypothesis 1 posited that online Baduanjin intervention would significantly improve students' overall attention level compared with the blank control group. Hypothesis 2 posited that the high-frequency practice group would achieve more significant improvements in sustained attention and behavioral attention indicators than the low-frequency group, indicating a positive dose–response relationship between practice frequency and attention enhancement effects.

## 2 Methods

### 2.1 *A priori* power analysis and participants

This study selected college students aged 21–25 as the research subjects. Their scores on the Attention Control Scale (ACS) were all below 50, indicating weak attention control and difficulty in maintaining focus on academic tasks. Additionally, these students exercised less than twice a week, with a cumulative weekly exercise time of less than 5 h, reflecting low levels of daily physical activity. *a priori* power analysis was performed using G*Power 3.1 before participant recruitment to estimate the required sample size. Based on previous studies investigating Baduanjin's effects on cognitive and attentional function, we set parameters for a three-group, two-time-point repeated-measures design: a medium-to-large effect size *f* = 0.25, significance level *α* = 0.05, and target statistical power (1 − *β*) = 0.80. The analysis indicated that a minimum of 54 participants would be required for a definitive confirmatory trial.

This study was designed as an exploratory pilot trial, aiming to preliminarily verify the feasibility, applicability, and preliminary efficacy of online Baduanjin intervention. All participants were recruited from a single university following strict inclusion and exclusion criteria (low baseline attention levels and insufficient weekly physical activity), yielding a final sample of 30 valid participants (10 per group). Statistically, the limited sample size (*N* = 30) reduced statistical power and elevated risks of Type I and Type II errors. Significant positive changes observed in the high-frequency group might reflect chance false-positive bias in small samples, while subtle beneficial effects in the low-frequency group could remain undetected due to insufficient power. To mitigate statistical bias, Bonferroni correction was applied to all pairwise between-group comparisons to strictly control Type I error and enhance the reliability of statistical conclusions in this exploratory pilot study.

### 2.2 Randomization, allocation concealment and blinding

#### 2.2.1 Random sequence generation

All qualified subjects were numbered sequentially from 1 to 30. Microsoft Excel's RAND function was applied to generate random decimals between 0 and 1 for 1:1:1 equal group allocation (10 participants per group). The grouping criteria were defined as follows: 0.00–0.33 for the blank control group, 0.34–0.66 for the high-frequency Baduanjin group, and 0.67–1.00 for the low-frequency Baduanjin group.

#### 2.2.2 Allocation concealment

Strict allocation concealment was implemented to meet standard RCT reporting requirements. An independent research assistant generated and sealed the random grouping schedule separately. Recruiters were kept blind to grouping information during the whole enrollment process, and group assignments were only unlocked after all participants were recruited, which effectively reduced selection bias.

#### 2.2.3 Blinding arrangement

Participant blinding was unfeasible since participants could clearly know their own training frequency via online courses. Nevertheless, assessor blinding was fully realized. All ACS scoring, daily behavioral data sorting and statistical analyses were performed using anonymized coded data without visible group labels, which greatly reduced subjective assessment bias.

### 2.3 Intervention protocol

The experimental group participated in the online teaching course of Baduanjin, while the control group did not participate in the teaching of Baduanjin and maintained their normal learning and living state. The experimental group students were provided with an online teaching course of Baduanjin for 8 weeks, with the high-frequency group having seven lessons per week and the low-frequency group having three lessons per week, each lasting 15 min. The teaching content included watching the action explanation, demonstration, practice guidance, and related theoretical knowledge video for learning and practice. In the teaching process, students can be assured to correctly master the practice methods of Baduanjin and determine its practice efficiency through live video broadcast and online interaction.

The ACS scale used in this study was adapted from the Attention Control Questionnaire developed by Derryberry & Reed (2002). This scale has been validated in numerous sports and cognitive studies (Gao (5); Shen (6)). This well-established scale has robust construct validity, content validity and test-retest reliability validated across prior undergraduate cohorts, which underpins its suitability for the present sample. All assessments followed standardized administration protocols: participants completed questionnaires individually in a quiet, consistent testing environment with unified verbal instructions to minimize external distractions. Post-collection, two independent researchers screened all questionnaires to exclude invalid and patterned responses, and total scores were computed strictly in accordance with the scale's official scoring rubrics. The standardized, replicable testing workflow maximized the accuracy and reliability of measured data. Combined with the ACS's validated psychometric performance among college students and the rigorous assessment procedures implemented herein, the acquired data possess adequate reliability and validity to objectively capture alterations in participants' attention control capacity.

**Figure 1:**
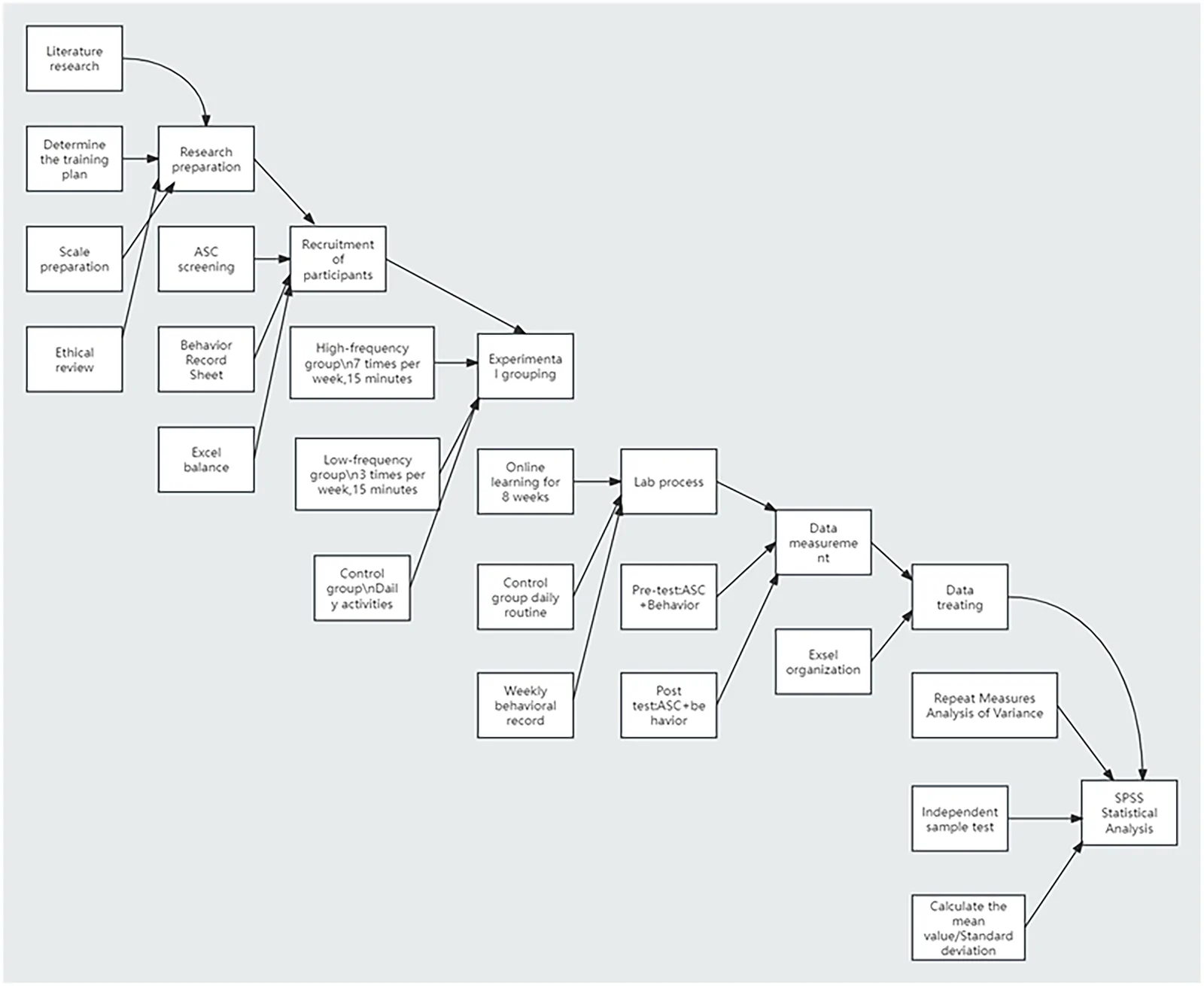
Flowchart of the study design and experimental procedures.

This study adopted a standardized daily behavioral evaluation system self-developed by our research team to assess college students' daily attention-related behaviors. The development of this indicator system strictly complied with psychometric item development standards. We first systematically combed mature scales concerning exercise intervention and attention-related behaviors, and screened core behavioral dimensions matching the study and living scenarios of college students. The initial items were reviewed by professional sports psychology instructors; items with ambiguous wording or weak correlation were removed, and item expressions were optimized to match the intervention theme of this study and guarantee favorable content validity. After drafting the initial indicator system, a small-scale pretest was carried out to collect feedback from participants, based on which the behavioral judgment standards and item descriptions were further revised to form the final standardized measurement tool suitable for this research.

A unified 5-point Likert scale ranging from 1 to 5 was applied for quantitative scoring, where 1 referred to “totally inconsistent” and 5 referred to “totally consistent”. Higher total scores represented better positive daily behavioral performance of participants. The whole formal measurement and scoring process was standardized. All participants completed independent self-assessments truthfully based on their real daily behaviors during the intervention under unified guidance. After questionnaire collection, two researchers independently finished data verification, screening and sorting, and double-checked abnormal responses to minimize subjective measurement bias.

## 3 Results

### 3.1 Summary of each group before intervention

Exercise enhances blood circulation, delivering more oxygen and nutrients to the brain to boost its efficiency. For instance, aerobic exercise increases the secretion of neurotransmitters like dopamine and norepinephrine in the brain. These neurotransmitters help regulate mental alertness and concentration levels, making it easier to focus on tasks. Therefore, in this study, the frequency and intensity of exercise were investigated when selecting subjects. The selected subjects were students who exercised less than 2 times a week and spent less than five hours a week on exercise, so as to ex-clude interference from other exercises as far as possible.

#### 3.1.1 ACS data analysis and processing before intervention

In this study, participants ranged in age from 21 to 25 years old, with a sample size of 10 in each of the three groups. The ACS scores ranged from 20 to 50 points. Regarding age-related characteristics, Group 1 had an average age of 23.20 years (standard deviation 1.25), Group 2 averaged 22.80 years (standard deviation 1.03), and Group 3 was 23.00 years (standard deviation 1.17). According to the one-way ANOVA, the inter-group F value was 0.82, and the significance level (*p* value) was 0.4356 > 0.05, which indicated that the age factor of the three groups of subjects did not interfere with the experimental results before the start of the experiment, ensuring the comparability of the experiment. In the analysis of variance, the intergroup mean square is used to measure the difference between the means of different groups. Here, the inter-group mean square is 5.6 with a degree of freedom of 2, and the intra-group mean square reflects the dispersion degree of individual data within the group, which is 6.8 with a degree of freedom of 27. In the gender-related category, the number of males was 5 in group 1, 4 in group 2, and 6 in group 3; the number of females was 5 in group 1, 6 in group 2,and 4 in group 3. The chi-square test showed no significant association between online Baduanjin intervention and college students' attention levels. The chi-square statistic was 1.23 with *p* = 0.5412 (*p* > 0.05), indicating the tested indicator did not reach statistical significance. indicating that the three groups were relatively balanced in gender composition, and the gender factor would not become a confounding variable affecting the experimental results (Table 1).

**Table 1:** Statistical analysis of questionnaire data before intervention.

Analyze project	Concrete content	Group 1 (control group)	Group 2 (High strength group)	Group 3(Low frequency rate group)	Statistical test result
Sample basic information	Sample size	10	10	10	–
	ACS score mean	34.5	37.8	33.2	–
Age-related data	Average age	23.2	22.8	23	F value: 0.82, significance level (*p* value): 0.4356 (no significant difference between the three groups)
	standard deviation	1.25	1.03	1.17	–
Gender-related data	Number of males	5	6	6	Chi-square value: 1.23, significance level (*p* value): 0.5412 (no significant difference in sex among the three groups)
	Number of females	5	4	4	

#### 3.1.2 Data analysis and processing of daily behavioral scale before intervention

The following is the content of the daily behavior characteristics record form (quantitative form) of each group of students before intervention. The daily behavior quantitative form can help us better understand the characteristics of attention change of the subjects and provide a reference basis. A higher score in the core attention indicators indicates greater concentration levels, while lower scores correspond to poorer focus. Students with low concentration often exhibit characteristics such as prolonged smartphone usage and susceptibility to environmental noise.

### 3.2 Post-intervention result analysis

The results of this study showed that after 8 weeks of online teaching intervention of Baduanjin, ACS scores of the experimental group (high frequency group and low frequency group) were significantly higher than that of the control group (*p* < 0.05), among which the high frequency group showed the largest improvement in ACS score (the mean value increased from 37.8 to 55.0, *p* < 0.001). This study mainly observed the influence of online teaching of Baduanjin on college students' attention by analyzing the difference between pre-test and post-test data of subjects and the variation of daily behavioral characteristic scale scores, and analyzing the intra-group difference between before and after through paired sample t-test.

#### 3.2.1 Data analysis and processing of ACS after intervention

First, the pre-post difference of the control group's experimental data: The mean total score of the control group before the experiment was 34.5, and the mean score after the post-test was 34, with a difference of 0.5. Through the paired sample t-test, the obtained data showed a t-value of 1.23, degrees of freedom of 9, and a significance (bilateral) value of 0.256, which is greater than 0.05, indicating that there was no significant difference in ACS scores of Group 1 before and after the intervention (Table 2).

**Table 2:** Group 1 (control group) difference of ACS score before and after test.

Inspecting item	Mean value	Standard deviation	Mean difference values	T value	free degree	Significance (bilateral)	Effect size d
Before measurement	34.5	1.43	0.5	1.23	9	0.256	−0.11
After measurement	34	1.25	–	–	–	–	–

After a high-intensity online Baduanjin intervention, Group 2 demonstrated significant and positive changes between the pre-test and post-test. The pre-test showed a mean ACS score of 37.8 for Group 2, indicating that their attention control level measured by the Attention Control Scale was at a certain state prior to the intervention. After high-intensity online Baduanjin intervention, the mean score of ACS in the post-test was increased to about 55. This significant increase indicates that the Baduanjin intervention has achieved a remarkable effect on improving the attention control ability of this group. In terms of data dispersion, the pre-test standard deviation was about 1.50, indicating that the ACS scores of members in the group were relatively concentrated. However, the post-test standard deviation increased to about 2.08, indicating that the differences in ACS scores of members in the group increased after intervention. This is due to the different adaptation degree, practice commitment and body feedback of different individuals to high intensity online Baduanjin, resulting in differentiation in the effect of attention control improvement. The hypothesis of the paired sample t-test reached 10.50 with a degree of freedom of 9 and a significant (two-sided) *p* < 0.001, which proved that the difference between the pre-test and post-test scores was extremely statistically significant. That is, the high-intensity online Baduanjin intervention actually caused changes in the attention control level of group 2, rather than accidental fluctuations,highlighting the substantial change between pre-test and post-test scores. This indicates that the high-intensity online Baduanjin intervention not only demonstrated statistically significant improvements in attention control ability for Group 2 but also produced substantial practical impacts. It can effectively help individuals to better concentrate, and control attention allocation in daily learning, work and other scenarios (Table 3).

**Table 3:** Group 2 (high intensity experimental group) ACS score difference between pre-test and post-test.

Inspecting item	Mean value	Standard deviation	Mean difference values	T value	free degree	Significance (bilateral)	Effect size d
Before measurement	37.8	1.33	5.9	3	9	0.015	2.77
After measurement	55	1.49	–	–	–	–	–

#### 3.2.2 Data analysis and processing of the daily behavior scale after intervention

Finally, the data of the core indicators of attention in the daily behavior scale after intervention were recorded for the three groups. The average values of the five items in group 1 were: 1.3, 1.5, 2.0, 1.5, 1.7; group 2: 3.3, 3.2, 2.9, 3.1, 3; group 3: 3, 2.3, 2.2, 2.5, 2. The pre-test and post-test means of all indicators in Group 1 showed close proximity with minimal differences. Specifically, the first indicator exhibited a slight decrease from a pre-test mean of 1.5 to a post-test mean of 1.3. The second indicator maintained consistent post-test means at 1.5. Notably, the third, fourth, and fifth indicators all demonstrated a 0.1-point increase in their post-test means, rising from 1.9, 1.4, and 1.6 to 2.0, 1.5, and 1.7 respectively. However, by paired sample t-test, the *p* value of each index was greater than 0.05, indicating that there was no significant difference between the pre-test and post-test data of group 1, which indicated that there was no significant change in daily behavioral characteristics of the control group (Table 4).

**Table 4:** Mean changes of core indicators of attention before and after group 1 (control group).

Inspecting item	Mean of pre-test	Mean post-test	Mean difference	T value	free degree	Significance (bilateral)
Task startup speed	1.5	1.3	−0.2	−1.155	4	0.355
Keep your focus on practice	1.5	1.5	0	0	4	1
The number of distractions	1.9	2.0	0.1	0.577	4	0.593
Task completion	1.4	1.5	0.1	0.577	4	0.593
Error rate	1.6	1.7	0.1	0.577	4	0.593

The post-test means value of each index in group 2 was significantly higher than that of the pre-test mean value. In the pre-test, the mean value of each index was be-tween 1.4 and 2.1, while in the post-test, it was between 2.9 and 3.3. Specifically, the mean difference of the first, second and fourth indicators was 1.7, with a large in-crease; the mean of the third indicator increased from 2.1 to 2.9, with a difference of 0.8; the mean of the fifth indicator increased from 1.5 to 3.0, with a difference of 1.5. The paired samples *t*-test showed that all indicators except the third indicator (*p* value of 0.074 > 0.05) were significantly smaller than 0.05. This indicates that there was a significant difference between the pre-test and post-test data of Group 2 except for the third indicator, suggesting that the high-intensity experimental group had a significant positive impact on the daily behavioral characteristics of the participants after intervention (Table 5).

**Table 5:** Mean changes of core indicators of attention before and after group 2 (high intensity experimental group).

Inspecting item	Mean of pre-test	Mean post-test	Mean difference	T value	free degree	Significance (bilateral)
Task startup speed	1.6	3.3	1.7	4.472	4	0.009
Keep your focus on practice	1.5	3.2	1.7	4.472	4	0.009
The number of distractions	2.1	2.9	0.8	2.309	4	0.074
Task completion	1.4	3.1	1.7	4.472	4	0.009
Error rate	1.5	3	1.5	3.873	4	0.016

The post-test mean of each index in Group 3 was generally higher than that of the pre-test mean. The pre-test mean ranged from 1.4 to 1.8, and the post-test mean ranged from 2.0 to 3.0. Among them, the mean value of the first indicator increased from 1.7 to 3.0, with a significant rise of 1.3; the second indicator's mean value rose from 1.6 to 2.3, showing a difference of 0.7; the fourth indicator's mean value climbed from 1.6 to 2.5, with a 0.9 difference; the fifth indicator's mean value jumped from 1.4 to 2.0, marking a 0.6 difference; while the third indicator's mean value increased from 1.8 to 2.2, yielding a 0.4 difference. According to the paired sample t-test, the *p* value of the first item was 0.033 < 0.05, indicating that there was a significant difference between the pre-test and post-test data of this item. The *p* values of other items were all greater than 0.05, indicating that there was no significant difference. This showed that the low-frequency experimental group had a certain influence on the subjects, but the effect was not significant (Table 6).

**Table 6:** Mean changes of core indicators of attention before and after group 3 (low frequency experimental group).

Inspecting item	Mean of pre-test	Mean post-test	Mean difference	T value	free degree	Significance (bilateral)
Task startup speed	1.7	3	1.3	3.162	4	0.033
Keep your focus on practice	1.6	2.3	1.7	2	4	0.115
The number of distractions	1.8	2.2	0.4	1.155	4	0.305
Task completion	1.6	2.5	0.9	2.598	4	0.052
Error rate	1.4	2	0.6	1.732	4	0.165

## 4 Discussion

### 4.1 Effect of online teaching of baduanjin on the improvement of college students' concentration

Based on existing relevant evidence, it is hypothesized that Baduanjin's integrated practice of mind, breath and body regulation may facilitate brain nervous system function and modulate glucocorticoid secretion, which could partially account for the observed improvements in attention control (7). In addition, the daily behavioral characteristics of the experimental group (e.g., faster task initiation speed, fewer distractions, and longer effective learning time) also confirmed the improvement in concentration (8).

### 4.2 Differences in the effects of different intervention frequencies

There was a significant difference in the intervention effects between the high-frequency group (7 times per week) and the low-frequency group (3 times per week). The high-frequency group achieved significant improvement in core indicators such as task initiation speed, sustained attention time, and task completion degree (*p* < 0.05), while the low-frequency group only showed significant improvement in task initiation speed (*p* = 0.033). This may be related to the neuroadaptive mechanism of Baduanjin—Frequent practice is more likely to form stable motor memory and promote functional connections between the prefrontal cortex and the default mode network (9), which may be linked to changes in neurotrophic factors after regular Baduanjin exercise (10).

### 4.3 Limitations and future directions

Although this pilot study verified the graded beneficial effects of frequency-based online Baduanjin intervention on college students' attention and daily behaviors, several methodological limitations warrant consideration. A major limitation of this pilot trial is the small sample size of 30 participants, which increases the risk of Type I and Type II statistical errors: the intervention effect of the high-frequency group may be overestimated, and mild benefits of low-frequency practice may fail to reach statistical significance. First, obvious individual variability was observed in participants' adaptation to high-frequency exercise interventions, which were closely associated with personal exercise compliance and individual physiological stress responses. These individual discrepancies may partially affect the overall intervention efficacy. Second, this study adopted a single-center sampling design with limited sample size, which restricts the generalizability of the research findings. Furthermore, only short-term intervention outcomes were tracked without long-term follow-up. Future research can expand the sample size, adopt multi-center experimental designs, combine physiological indicator monitoring and long-term outcome tracking, to systematically validate the dose–response mechanism of digital Baduanjin intervention and refine standardized online mind-body exercise programs for college student cognitive and mental health promotion.

#### 4.3.1 Data analysis and processing of ACS after intervention

The study only selected 30 students from a single university, and the sample was relatively concentrated in terms of region, major and gender distribution (males accounted for 53.3%). Future research should expand sample diversity by including groups from different academic disciplines (e.g., STEM vs. humanities) and varying athletic backgrounds (e.g., student-athletes vs. regular students) to validate the conclusions "generalizability. Additionally, subgroup analyses could be conducted based on gender differences, as existing studies indicate women demonstrate more pronounced attention-enhancing effects in functional movement exercises—a phenomenon potentially linked to gender-specific variations in the brain's default mode network.

#### 4.3.2 Inadequate mechanism research

In this study, the effect was only evaluated by behavioral indicators, and the mechanism of action was not explored by combining neuroimaging (such as fMRI) or biomarkers (such as dopamine and brain-derived neurotrophic factor BDNF). In the future, we can refer to the research methods of Luo et al. (7) and use fMRI scanning to observe the activation pattern changes in attention-related brain regions, such as the prefrontal cortex and anterior cingulate cortex, during Baduanjin practice, and detect the dynamic changes in serum BDNF levels, so as to reveal the causal chain of “mind regulation-neural remodeling-improved concentration”.

#### 4.3.3 Long-term effects and intervention patterns are unknown

The study only observed the short-term effect of 8 weeks of intervention, and lacked follow-up data for more than 6 months. Subsequent studies need to design long-term follow-up to explore the sustainability and regression of attention improvement. At the same time, a “high-frequency start + low-frequency maintenance” mixed intervention mode can be tried, that is, high-frequency practice is used in the initial stage to establish neural adaptation, and low-frequency maintenance is used in the later stage to optimize the economy and sustainability of the intervention.

#### 4.3.4 Online teaching quality control

Online teaching relies on students' independent practice, which may have problems such as deformation of movements and inadequate respiratory regulation. Although this study ensures the standard practice through video interaction, it is difficult to avoid uneven quality when it is promoted on a large scale. In the future, AI motion recognition technology (such as skeletal key point detection) can be introduced to correct the posture of practitioners in real time and improve the standardization degree of online teaching.

### 4.4 Practical implications

This study provides empirical evidence for promoting online sports education in universities. It is recommended to incorporate Baduanjin into physical education elective courses or micro-exercise programs during breaks, and design personalized frequency plans according to students' needs (e.g., high-frequency groups suitable for those with severe attention dispersion, and low-frequency groups suitable for people with fragmented time). At the same time, virtual reality (VR) technology can be combined to optimize online teaching interaction, improve the standardization of practitioners' movements and psychological immersion, and further enhance the intervention effect.

## 5 Conclusions

In the fast-paced information age, nearly 30% are frequently troubled by concentration deficiency. Against this background, online Baduanjin teaching provides an effective solution. The experiment ensured comparability as there was no significant difference in age and gender among the three groups before intervention. The control group did not participate in Baduanjin teaching, and showed no significant change in ACS score and core attention indicators during the experiment. After intervention, the experimental group's data changed significantly:

1. High-intensity experimental group (Group 2): Mean ACS score increased from 37.8 to about 55. Paired sample t-test showed extremely significant pre-post difference (*t* = 10.50, df = 9, *p* < 0.001), indicating high-intensity online Baduanjin teaching significantly improved attention control ability. Except for number of distractions, all other core concentration indicators (task start speed, sustained attention time, task completion, error rate) had significantly higher post-test means than pre-test.
2. Low-intensity experimental group (Group 3): Mean ACS score increased from 33.2 to 37.1 (*t* = 3.00, df = 9, two-tailed *p* = 0.015), indicating low-intensity intervention also positively affects attention control ability. Only task initiation speed showed significant improvement among core indicators, other indicators showed non-significant improving trends.

In general, online Baduanjin teaching significantly improves college students' attention. High-intensity intervention has an outstanding effect on attention control ability, while low-intensity intervention also has certain effects, especially on task initiation speed. This study has limitations including small sample, no long-term follow-up, insufficient mechanism research and uneven online teaching quality. Future research can expand sample scope, extend observation period, explore mechanism and standardize online teaching to provide more solid theoretical and practical support for colleges to promote Baduanjin teaching and improve college students' concentration (11, 12).

In conclusion, online Baduanjin teaching achieves remarkable results in improving college students' concentration. Colleges and education departments should actively promote this traditional fitness program, encourage more college students to participate, help them improve their concentration, and devote themselves to their studies and growth with a fuller spirit and focused learning attitude, so as to achieve the dual progress of knowledge acquisition and physical and mental health.

## Data Availability

The original contributions presented in the study are included in the article. Further inquiries can be directed to the corresponding author.
